# Editorial

**Published:** 1849-06

**Authors:** 


### THE MEDICAL EXAMINER.

PHILADELPHIA, JUNE, 1849.

### AMERICAN MEDICAL ASSOCIATION.

*Second Annual Meeting.*

The Association met in Boston, May 1st, in the Hall of the Lowell Institute.

The President, Dr. Alexander H. Stevens, of New York, in the chair.

Dr. J. C. Warren, of Boston, on behalf the Massachusetts Medical Society, presented to the Association the salutation and welcome of his constituents; after which an address was delivered by Dr. Stevens, in which he dwelt upon the objects and duties of the Association, the advantages already derived from it, by the partial reforms which had been commenced in accordance with its recommendations; as well as the great good which must necessarily follow from promoting personal intercourse among members of the profession from various parts of the country, and thus ensuring interchange of sentiments and opinions at stated periods.

The committee of arrangements reported twenty States represented, and two hundred and eight delegates, as registered at ten o'clock the evening previous. The number present, as afterwards reported, was about four hundred and fifty.

The committee on nominations, consisting of one from each State, appointed by the delegates, reported the following officers for the ensuing year, and their nomination was unanimously confirmed. *President,* Dr. J. C. Warren, Mass.; *Vice Presidents,* Dr. J. P. Harrison, Ohio, Dr. H. H. Maguire, Va., Dr. A. Flint, N. Y., Dr. R. S. Stewart, Md.; *Secretaries,* Dr. Alfred Stillé, Pa., Dr. H. J. Bowditch, Mass.; *Treasurer,* Dr. I. Hays, Pa.

The committee on nominations were continued, and instructed to nominate the usual standing committees for the ensuing year; and to facilitate their operations, it was requested that the names of suitable persons to act upon these committees should be handed in.

Dr. Condie, of Philadelphia, chairman of the committee on Practical Medicine, presented the annual report. After reading a small portion of it he was interrupted by a motion to refer it to the committee on publication, which after some discussion was carried.

[We cannot refrain from commenting upon the impolicy, to say nothing of the discourtesy, of such a proceeding as this. By establishing a precedent of this kind, one great motive for thus assembling from the distant portions of the Union is abolished, viz: the interchange of sentiments and opinions that arise from the discussion of the reports. A prominent feature in the organization of the Association seems to have been lost sight of, to wit, the advancement of the knowledge, and the extension of the usefulness of the profession at large.

If the whole object of the Association be to appint committees whose reports shall be printed in the transactions without comment, this can be accomplished without the sacrifice of time that now takes place in travelling from remote portions of the country.

Aside from all this, there is a *discourtesy* in thus silencing those who have spent much valuable time in the diligent search after material wherewith to enrich those reports, and it offers but little encouragement to future committees, when they see the labours of their predecessors so coldly received. Neither do we think the matter was mended by the proposition to refer the reports to the committee on publication, previously to their being read in the Association, that they might select such parts as seemed most important, and omit the rest, and thus "cut out" a sufficient amount of work to occupy the Association during a specific season. There are few authors, we imagine, who would quietly submit their bantlings to the unmerciful pruning of a publishing committee, in order that time may be allowed for the effervescence of gaseous debaters. We are credibly informed that several original and important communications were withheld in consequence of the course adopted in relation to the committee on Practical Medicine. It is to be hoped, therefore, that if committees are appointed in the spirit of the organization of the Association, that in future meetings they will be heard with respectful attention.]

Dr. N. R. Smith, of Baltimore, presented and read the annual report of the committee on Surgery. A large portion of the report was occupied in the consideration of anæsthetic surgery, to which it was entirely favourable. The committee consider it inadmissable to perform a serious surgical operation without the use of chloroform, inasmuch as by it both safety and immunity from pain are secured. Of the two prominent anæsthetic agents, chloroform and ether, the former is preferred, inasmuch as its unfavourable effects, when they do occur, are visible at once, whereas, when ether is used, its consequences sometimes remain long after. Dr. S. thinks he has traced irritative fever to the protracted influence of the latter agent.

Chloroform is the most powerful anæsthetic agent known, and requires that care should be used in its administration. It should never be used in trivial cases, nor in diseases of the heart: a due admixture with atmospheric air is also requisite for safety. In careful hands it is an invaluable agent. The author of the report has administered it *thirty-four* times to one patient, a young woman, to the extent of complete insensibility, without any unpleasant result. Prof. Mott, of New York, has performed operations which he would not have attempted without the aid of chloroform. In the administration of it, it should be stopped the moment that insensibility occurs. Prof. Simpson has published his opinion that one hundred lives have been preserved by the use of chloroform, where one has been lost by its use. He further states, that the mortality where chloroform is used, is much less than in similar cases where its use is dispensed with.

On the subject of Fractures, the report was also voluminous; this department was occupied mainly, however, with the exhibition and explanation of an apparatus for the treatment of fractures of the lower extremities, invented by the chairman. As this has been known for some years to the profession, and has been used in many of our public institutions, a description of it is unnecessary. The same is true of the instrument exhibited for the operation of lithotomy. The latter is, moreover, open to the objection that it almost supersedes the necessity for a correct knowledge of surgical anatomy.

The entire report was listened to with marked attention, and referred, without discussion, to the committee on publication.

Dr. C. R. Gilman, of New York, acting chairman, presented and read the annual report of the committee on Obstetrics.

The greater part of this report was also occupied in the discussion of anæsthetics in midwifery; and in order to present the subject candidly to the Association, the principal objections of those opposed to its use were incorporated into the report. The committee give it as their deliberate opinion, that the chances of a patient's recovery are greatly increased by the use of anæsthetics, and the question is not whether they may or may not be safely administered, but whether they *can be rightfully withheld.* Who that has ever compared the panting and exhausted subject of an instrumental labour, with the calm and tranquil recipient of anæsthesia, could fail to arrive at the same conclusion? In regard to the choice of anæsthetics, the report declares that chloroform has every advantage over ether, except *in point of safety*, but that in experienced hands this objection does not obtain. Dr. Channing's contribution to the literature of this subject, in his work "Etherization in Child-birth," received a justly deserved and flattering notice in this portion of the report. The report was accepted, and referred without comment to the committee on publication.

Dr. J. P. Harrison, of Ohio, presented and read the annual report o the Committee on Medical Literature. The report embraced the division marked out by the constitution, viz:

*First.* The general character of medical periodical literature in the United States.

*Second.* A consideration of the most important and prominent articles that are thus brought to our notice.

*Third.* Original or native American medical publications.

*Fourth.* Medical compilations and compends of American writers.

*Fifth.* American reprints of foreign periodical medical books.

*Sixth.* All such measures as may be deemed advisable for encouraging and maintaining a medical literature of our own.

Under the first head, the report states that there are twenty American medical journals published in the United States, and four reprints of foreign journals. Of these, five are quarterlies, six are issued bimonthly, six monthly, one three times a year—the Transactions of the Philadelphia College of Physicians—and one weekly.

Through the agency of these journals much valuable material has been added to the medical literature of the country, and their pages have been enriched by contributions from the most eminent members of the profession both at home and abroad.

Of the American contributions a brief summary was embraced in the report.

The library of the Pennsylvania Hospital was described as the largest in the country. Commencing originally in 1762 by the donation of a single volume by Dr. Fothergill of London, it has increased to the extent of ten thousand volumes. There are many other extensive libraries throughout the country, some containing seven thousand, others three thousand, and two thousand volumes. The Library of Harvard College numbers one thousand seven hundred and sixty-nine volumes; that of the medical department of Harvard University twelve hundred volumes.

That portion of the report embracing the measures deemed advisable for encouraging and maintaining a medical literature of our own, stated that there was much valuable literary material unknown to the public in consequence of deficient means on the part of the authors, or a disinclination on the part of publishers to take hold of anything that was not endorsed by a well known name, and instanced the unpublished literary remains of the late Dr. Forry, of New York. It recommended that a board of publication should be established, to whom such materials should be presented, with authority to publish them should they be deemed worthy.

Appended to the report was the following resolution:

*Resolved*, That a committee of three be appointed to take into consideration the measures recommended in this report, for the promotion of our national medical literature, with instructions to report at the next annual meeting.

The report of the Committee on Medical Literature was accepted, and referred to the committee on publication, and the resolution appended to it was adopted; Drs. Horner, Condie, and Hays, of Philadelphia, were appointed the committee.

Dr. G. B. Wood, of Philadelphia, moved that it be the duty of the same committee to report on the subject of an international copy-right law.

In urging this motion Dr. Wood remarked, that it was essential to the medical literature of the country, that an international copy-right law be established. He claimed it for our own writers, who now receive no encouragement. American publishers can now procure and reprint foreign books for a less price than American authors can afford to write them. They must produce a better book, a great deal better book than the English writer, or they cannot find an American publisher who will pay them for their work. He claimed it also on the ground of justice to English writers, who were despoiled of the labour of their head and hands by the cupidity of our booksellers.

The motion was *carried*.

Dr. M. L. Taft, on behalf of Dr. F. C. Stewart, of New York, chairman of the Committee on Medical Education, presented and read the annual report, which, in accordance with the requirements of the constitution, embraced a complete account and comparison of the medical institutions of Europe and this country, with the requirements for admission and graduation; the number of students, graduates, professors, branches taught, terms of study, &c.; the regulations and requirements of Army and Navy Boards of Examiners in Great Britain and this country; the legal requirements exacted of medical practitioners in the several States of the Union; together with remarks on the general condition of medical education in the United States, compared with other countries, with suggestions as to its improvement.

In the comparison of schools abroad and at home, the University of Pennsylvania was held up as the model for imitation in the United States, that institution being the oldest, and coming nearest to the standard deemed most desirable. The report further stated that to the inquiries addressed to the thirty-seven medical schools in the United States, in relation to the requirements for admission, graduation, &c., answers have been received from twenty-five.

With regard to the best method of improving medical education, the report recommended the insisting upon preliminary education, and the appointment of primary boards of examiners, whose certificate of qualification shall be essential to the reception of the student into a medical school. The board should examine the candidate (should he not be a graduate of some literary institution) upon Latin and Greek, and require suitable testimonials as to moral character. The subject of the extension of the lecture term was not alluded to in the report, probably because the Association had already settled that question affirmatively.

Dr. John Ware, of the Medical department of Harvard College, presented a paper in answer to the queries of the Committee on Education, from a Committee of the Faculty who were appointed to take into consideration some of the recommendations of the Medical Association with regard to medical lectures, particularly in reference to extending the courses of lectures beyond the established period of four months. The purport of the paper was that the Faculty were constrained to differ from the views of the Association with regard to the prime importance of lectures, and also that in their view no profitable object could be gained by extending the term of lectures beyond a period of four months. Lectures are a subordinate and subsidiary part of a medical education. The great object in view from them is to teach the student how to study for himself. The paper did not undervalue the importance of medical lectures—far from it. Information was communicated through these sources which would not be accquired any other way, but it was desirable that they should take their proper place in the education of students. It regarded the establishment of private Medical Schools in our cities as of very great importance.

A series of resolutions was appended to the report, upon which con siderable discussion arose. The report of the committee was accepted and referred to the committee on publication, and the resolutions were brought separately before the Association in committee of the whole.

The following are the resolutions which were appended to this report:

1st. *Resolved,* That the attention of Medical Colleges be again directed to the resolutions of the Committee on Preliminary Education adopted by the Medical Convention of 1847, and that they be advised to require from students that they shall in all cases produce certificates of preliminary education. *Carried.*

2d. *Resolved*, That the several State and County Societies, as well as all voluntary Medical Associations throughout the country, be advised and requested to adopt the plan proposed by the Medical Society of the State of New York, at its last annual meeting, for ensuring due attention to the subject of preliminary education.

Dr. Davis, of New York, explained that the plan of the New York State Society was, that every County Society should appoint a board for preliminary examinations of students, with a view that they should be required to produce certificates from such boards before they could be received as medical students in the office of any private medical practitioner.

A gentleman, whose name we could not learn, offered the following as an amendment:

*Resolved*, That as students are generally introduced to the profession by private preceptors, it is recommended that no student be received by them unless they come up to the standard of preliminary education prescribed by this Association.

The question being upon the amendment, it was adopted by a large vote.

3d. *Resolved*, That this Association does not sanction or recognize "College clinics" as *substitutes* for Hospital clinical instruction, and that the Medical Colleges be again advised to insist, in all instances where it is practicable, on the regular attendance of their pupils during a period of at least six months upon the treatment of patients in a properly conducted Hospital or other suitable institution devoted to the reception and care of the sick.

The resolution was adopted.

4. *Resolved*, That it would conduce both to the convenience and advantage of students, if the subjects taught in the Colleges were divided into two series; the one of which should be studied during the first year's attendance on lectures; and the other, during the second session. And that examinations should be substituted at the close of the first course of lectures on the subjects taught during that course, certificates of which should be required prior to the final examination. Rejected.

5. *Resolved*, That it is the deliberate opinion of this Association that the plan of examining students for medical degrees in private, and before one professor only at a time, is highly defective, and should be at once discontinued. Laid upon the table.

6. *Resolved*, That examinations for medical degrees should be practical, and that it is desirable as far as practicable that they should be conducted in writing as well as *viva voce*. Laid on the table.

7. *Resolved*, That in view of the importance of a due knowledge of practical pharmacy, the medical schools be advised to require from candidates for degrees that they should produce satisfactory evidence of their having been engaged in compounding medicines and putting up prescriptions, either under the direction of their private preceptors, or in the shop of a recognized and qualified apothecary. Laid upon the table.

In regard to examining boards and licenses:

8. *Resolved*, That the interests both of the public and the medical profession would be promoted by the establishment of boards of examiners in each of the States of the Union, to examine candidates for licenses to engage in the active practice of medicine and surgery. Laid upon the table by a vote of 69 to 54.

9. *Resolved*, That the standard of requirements established by the examining boards of the several states should be uniform, and that the examinations should, as far as practicable, be conducted in a similar manner. Laid upon the table.

10. *Resolved*, That the examiners should, in all instances, satisfy themselves that candidates are familiar with the elementary branches of general knowledge. Laid upon the table.

11. *Resolved*, That for the purpose of carrying out the objects contemplated in the foregoihg resolutions, a special committee of seven members be appointed to prepare a memorial and form of law in reference to the subject of the establishment of boards of medical examiners to be submitted to the Association at its next annual meeting. Indefinitely postponed.

The committee of the whole having thus considered the resolutions submitted to them by the report on medical education, arose and reported to the Association, and their action was confirmed.

On motion of Dr. Stevens, of New York, it was voted, That the whole subject matter of medical education, together with the resolutions which have been passed, and those which have been laid upon the table, be referred to a special committee of three members, with instructions to report to-morrow morning. The chair appointed Dr. Stevens, of New York, Dr. Wood, of Philadelphia, and Dr. Knight, of Connecticut, as the committee.

The following is the report of that committee:

1st. *Resolved*, That the Association reiterate their approval of the resolutions in reference to medical education, adopted by the Convention, which met in Philadelphia, in May, 1847, and contained in pages 73 and 74 of the published proceedings of that Convention.

2d. *Resolved*, That the attention of Medical Colleges be again directed to the Resolutions of the Committee on Preliminary Education, adopted by the Medical Convention of 1847, and that they be advised to require from their students that they shall, in all instances, present certificates of due preliminary acquirements prior to graduation.

3d. *Resolved*, That physicians, generally, throughout the Union, be advised and requested to require of those wishing to become their pupils, evidence of a proper general education, before admission into their offices.

4th. *Resolved*, That the Association does not sanction or recognise "College Clinics" as substitutes for Hospital clinical instruction, and that the Medical Colleges be again advised to insist, in all instances, where it is practicable, on the regular attendance of their pupils, during a period of six months, upon the treatment of patients in a properly conducted hospital, or other suitable institution, devoted to the reception and cure of the sick.

5th. *Resolved*, That in accordance with a resolution of the American Medical Association, adopted May 4th, 1847, "it is earnestly recommended to the physicians of those States in which State Medical Societies do not exist, that they take measures to organise them before the next meeting of this Association."

6th. *Resolved*, That the State Societies be recommended, after they shall have been organised, to recognise as regular practitioners none who have not obtained a degree in medicine, or a license from some regular medical body, obtained after due examination.

7th. *Resolved*, That the Association recommend to the various Schools of Medicine to meet at Cincinnati before the next annual meeting of this Association, and present a plan for elevating the standard of medical education to this Association.

The committee do not deem it expedient that the Association should now adopt, further than may have been done in the preceding resolutions, the recommendations offered in the several documents referred to them.

On motion of Dr. Harrison the report was accepted, and the Association went into Committee of the whole, Dr. R. D. Arnold in the chair, for the purpose of considering the resolutions attached to the report. These were taken up successively, and, after prolonged discussion; were reported to the Association without amendment, but with the addition of the following, proposed by Dr. T. E. Bond, Jr., of Maryland.

*Resolved*, That this Association recommend the encouragement of private medical institutions, strongly advising that Dispensary practice be made, as far as practicable, a part of the means of instruction.

The resolutions reported by the Committee of the whole were then adopted.

The annual report of the Committee on Hygiene, Dr. Jas. Wynne, of Baltimore, Chairman, was, in his absence, presented and read by Dr. Isaac Parrish, of Philadelphia.

Appended to this report were two able and interesting papers, one presented and read by Prof. S. Jackson, of Philadelphia, on the influence of tea and coffee used as food; and one by Dr. Josiah Curtis, on the Sanitary Condition of Massachusetts.

The report and the papers were accepted, and referred to the Committee on Publication.

The annual report of the Committee on Indigenous Botany was presented by the Chairman, Dr. N. S. Davis, of New York. The report (of which a verbal synopsis only was given) stated that our acquaintance with the medicinal properties of our indigenous plants was very slight and unsatisfactory. The committee, during the past year, had been making careful investigations, both by analysis and experiment, to discover the actual value and precise action of a number of substances admitted into the materia medica, concerning which the books gave no satisfactory account. As illustrative of this, he stated that of 1000 plants, reputed to possess medicinal virtues, but 150 are even slightly known. Of 280 native and naturalized plants mentioned in one of our best works on botany, we are told, concerning 150 of them, merely that they have been employed by the Indians for such and such purposes. This kind of information was not such as the present state of scientific accuracy demanded. Very little is known of the real virtues and uses of our native plants, but it is hoped that the investigations which have been commenced, under the auspices of the Association, will be continued and perfected.

The report was accepted and referred to the Committee on Publication.

The Committee appointed to consider the subjects presented by the report on Medical Literature, and the resolution of Dr. Wood, obtained permission to report *in part*, and submitted the following resolution.

*Resolved*, That a committee of three be appointed to memorialise Congress in favour of an international copy-right law.

This was so far amended as to require the proposed committee to prepare a memorial upon the subject, and submit it to the Association at its next annual meeting. The motion as amended was *adopted*, and Drs. G. B. Wood, T. E. Bond, I. Hays, were appointed as the committee.

The following preamble and resolution, presented by Dr. Evans, of Indiana, were adopted.

*Whereas*, Merit should be the test by which one individual is preferred to another; and, *whereas*, the places of profit and honour in our profession should be open to the competition of all, in order that the best selections may be made, therefore,

*Resolved*, That Trustees and others, exercising the office of appointing Professors in Medical Schools, be requested to adopt the system of *concours*, or public trials, among the means resorted to for calling out the talent of the profession, and ascertaining the qualifications of applicants.

Dr. G. B. Wood submitted the following preamble and resolution, which were adopted.

*Whereas*, A document prepared by the Medical Faculty of Harvard University, and appended to the Report of the Committee on Medical Education, contains an elaborate defence of the limitation of the courses of medical instruction in the schools to four months; and, *whereas*, this document has been referred, along with the Report of the Committee on Medical Education, to the Publishing Committee, and, if it be not mistaken by the public as a representation of the views of this Association, may, at least, have the effect of contravening those views, unless they be properly supported; therefore,

*Resolved*, That a committee be appointed to prepare, at leisure, a statement of the facts and arguments which may be adduced in favour of the prolongation of the courses to six months; and that the statement thus prepared be printed in the forthcoming volume of the

Transactions of the Association. Drs. S. Jackson, (Prof.); J. L. Atlee, and A. Stillé, were appointed the committee.*

Dr. U. Parsons, from a Select Committee appointed at the meeting of 1848, made a report on the subject of adulterated and spurious drugs, and offered the following resolutions.

*Resolved,* That a committee, consisting of two delegates from each State here represented, be chosen by the President, to note all the facts that come to their knowledge with regard to the adulteration and sophistication of drugs, medicines, chemicals, &c., and to report them at the next annual meeting.

*Resolved,* That the President be requested to sign, and forward to the Philadelphia College of Pharmacy, a letter, stating that the Association are pleased to hear of its laudable intention to prepare and publish some simple directions for detecting adulterations in medicines, adapted to the understanding of the people generally, and would be highly

* In a notice of the proceedings of this meeting of the Association, in the Boston Medical and Surgical Journal for May 16, the author remarks as follows, in relation to this resolution of Dr. Wood.

"It has been thought that this resolution, as it appears in the Transactions of the Association, will bear upon it the endorsement of the Association. It is obvious it will do this no more than will Dr. Ware's paper, which we have seen has been appended to the report on Education. They simply state the opinions of members of medical faculties in Pennsylvania and Massachusetts, and for which the Association is in no sense responsible."

The paper presented by Dr. Ware was prepared in answer to the queries of the Committee on Education, and delivered to them after the Association had met, and was permitted to be read in connection with their report. By an act of courtesy, this paper, which was in direct opposition to the published views of the Association, was admitted into the Transactions.

It was in order to counteract the opinion that might arise from seeing this paper in the published Transactions, that the Association had undone all that it had formerly decided upon in relation to the six months course, that Dr. Wood's resolution was offered, and a committee appointed to prepare a statement of facts and arguments in favour of the prolongation of the lecture term, to be published along with Dr. Ware's paper.

This committee, then, which was appointed *to sustain the views of the Association* in relation to a matter upon which it had already decided three times, *will,* most certainly, bear upon it the endorsement of the Association, so far as the expression of its opinions goes. All that it shall do, in the legitimate performance of its duties, will be by the authority of the Association, and cannot, therefore, be considered as a mere expression of the opinions of members of medical faculties in Pennsylvania.—Eds. Ex.

gratified could they welcome its appearance before the next annual meeting.

Dr. Ware submitted the following resolution, which was adopted.

*Resolved*, That the Committee on Practical Medicine be instructed to inquire into the expediency of adopting the English language exclusively in the writing of prescriptions, and in all directions for the composition and administration of medicines, and to report at the next annual meeting of the Association.

Dr. G. B. Wood, of Philadelphia, stated that he had a brief report to make, as a delegate from this Association to the British Association, and to the Provincial Medical and Surgical Association of England, the annual meetings of which he had attended in August last, in fulfilment of the objects of his appointment. Of the British Association he had only to say, that he was treated with all personal courtesy, and invited to participate in the proceedings of that body with the privileges of a member. By the Provincial Medical and Surgical Association he had been received with the most flattering distinction in his capacity as a delegate. The Association appeared to be much gratified by the compliment paid them, and expressed, through their President, their high appreciation of this Society, and their reciprocation of the sentiments conveyed to them; and passed a resolution, unanimously, requesting him (Dr. Wood) to convey their thanks to the American Association.

Dr. J. B. Johnson, of Missouri, introduced the following preamble and resolution, originally presented by him to the Medical Convention at Philadelphia, in 1847, and they were referred to the Committee on Medical Education.

*Whereas*, Numberless and important evils result from the almost universal practice of allowing persons, wholly ignorant of drugs and medicines, to engage as Apothecaries; and still greater, from the universal traffic in patent and secret remedies; therefore,

*Resolved*, That the Committee on Education inquire into the expediency of establishing a school or schools of Pharmacy in the respective States, for the special purpose of preparing persons for the business of Apothecaries; and also the expediency of adopting a rule, that no Physician ought to patronise a Druggist or Apothecary who deals in patent and secret medicines—and report at the next annual meeting of the Association.

Dr. James Wood, of Pennsylvania, presented the following resolution, which was *adopted*.

*Resolved*, That the Committee on Medical Science for 1849, be instructed to inquire into the expediency of establishing a Board to analyze the quack remedies and nostrums now palmed upon the public, and to publish the results of their examinations in a newspaper to be established for the purpose; and farther, to append such plain views and explanations thereto as will enlighten the public in regard to the nature and dangerous tendencies of such remedies.

Dr. Stevens, of New York, offered the following resolution.

*Resolved*, First, that a committee of seven be appointed to consider the subject of forensic medicine; second, a similar committee on indigenous botany and materia medica; and third, a committee on hygiene;—the committees to be nominated by the general Nominating Committee. Carried.

The Committee on Nominations reported the following Standing Committees to act for the ensuing year. *Adopted.* The list is as corrected by the Nominating Committee, after the vacancies created by resignation had been filled.

*Committee on Medical Science.*

Dr. Usher Parsons, Providence, R. I., Chairman.

| | |
|---|---|
| Dr. J. Bigelow, Boston. | Dr. Jas. Moultrie, Charleston, S. C. |
| " J. B. S. Jackson, Boston. | " G. Emerson, Philadelphia. |
| " A. B. Malcolm, Dubuque, Iowa. | " D. King, Newport, R. I. |

*Committee on Practical Medicine.*

Dr. J. K. Mitchell, Philadelphia, Chairman.

| | |
|---|---|
| Dr. R. La Roche, Philadelphia. | Dr. B. R. Jones, New Orleans. |
| " F. West, Philadelphia. | " R. D. Arnold, Savannah. |
| " J. A. Swett, New York. | " ——— Smith, Indiana. |

*Committee on Surgery.*

Dr. R. D. Mussey, Cincinnati, Chairman.

| | |
|---|---|
| Dr. W. M. Awl, Columbus, Ohio. | Dr. L. A. Dugas, Augusta, Ga. |
| " A. B. Shipman, Syracuse, N.Y. | " S. Parkman, Boston. |
| " G. Fox, Philadelphia. | " J. R. Wood, New York. |

*Committee on Obstetrics.*

Dr. T. G. Prioleau, Charleston, S. C., Chairman.

| | |
|---|---|
| Dr. L. D. Ford, Augusta, Ga. | Dr. H. F. Askew, Wilmington, Del. |
| " Robert Lebby, Charleston, S. C. | " John Evans, Chicago, Ill. |
| " Josiah Bartlett, Stratton, N. H. | " Isaac Lincoln, Brunswick, Me. |

*Committee on Medical Education.*

Dr. J. Roby, Baltimore, Md., Chairman.

Dr. Blatchford, Troy, N. Y.

" G. M. C. Roberts, Baltimore.

" R. W. Sylvester, Norfolk, Va.

Dr. F. A. Ramsay, Knoxville, Tenn.

" Geo. Sumner, Hartford, Conn.

" W. F. Rockwell, Brattleboro, Vt.

*Committee on Medical Literature.*

Dr. Alfred Stillé, Philadelphia, Chairman.

Dr. F. G. Smith, Philadelphia.

" T. H. Yardley, Philadelphia.

" P. C. Gaillard, Charleston, S. C.

Dr. N. T. Morris, Montgomery, Ala.

" J. Fithian, Woodbury, N. J.

" J. B. Johnson, St. Louis, Mo.

*Committee on Publication.*

Dr. I. Hays, Philadelphia, Chairman.

Dr. A. Stillé, Philadelphia.

" H. J. Bowditch, Boston.

" D. F. Condie, Philadelphia.

Dr. B. T. Barker, Norwich, Conn.

" Isaac Wood, New York.

" M. J. Pittman, Rocky Mt., N. C.

*Committee on Forensic Medicine.*

Dr. A. H. Stevens, N. Y., Chairman.

Dr. Luther V. Bell, Boston.

" Pliny Earle, New York.

" W. F. Rockwell, Vt.

Dr. Robert Watts, New York.

" R. S. Stewart, Baltimore.

" J. Knight, New Haven, Conn.

*Committee on Indigenous Botany and Materia Medica.*

Dr. Eli Ives, New Haven, Chairman.

Dr. G. L. Corbin, Warwick Co. Va.

" H. R. Frost, Charleston, S. C.

" W. H. Davis, Baltimore.

Dr. B. B. Lenoir, Roane Co. Tenn.

" W. B. Cochran, Middleburg, Va.

" J. P. Harrison, Cincinnati.

*Committee on Hygiene.*

Dr. J. M. Smith, N. Y., Chairman.

Dr. A. K. Gardener, New York.

" E. Jarvis, Dorchester, Mass.

" A. G. M. Cooke, Norfolk, Va.

Dr. A. S. Holmes, St. Louis, Mo.

" G. Emerson, Philadelphia.

" J. C. Simonds, New Orleans.

The committee recommended CINCINNATI as the next place of meeting, and the following as the Committee of Arrangements:—Dr. Dodge, Dr. Judkins, Dr. Rives, Dr. Lawson, Dr. Richards and Dr. Strader, all of Cincinnati.

On motion a vote of thanks was presented to the Officers of the Association for the efficient and courteous manner in which they had discharged their duties, and to the Committee of Arrangements for their kind and hospitable reception of the delegates. Dr. Z. B. Adams responded on behalf of the Committee of Arrangements; and, after receiving the congratulations of the President upon the happy termination of their labours, the Association adjourned on Friday evening, May 4th, *sine die.*

We have thus endeavoured to present to our readers a condensed account of the proceedings of the Association, derived from the various reports of the daily press, and from notes taken on the spot by one of the Editors.

To all engaged in it we are sure the meeting was a pleasurable one, and indicated a growing interest in the means proposed to elevate the standard of the profession. The number of delegates was very much larger, and the session protracted to a greater length, than on any previous occasion, and yet nothing occurred to mar the harmony of the meeting; a conciliatory spirit seemed to pervade the whole proceedings. The brief analysis of the Reports, given above, is but a feeble expression of their merits; we trust, however, at some future time, to do them more justice. The only regret experienced in relation to them was, in reference to their curtailment, on which we have already commented.

To the Physicians of Massachusetts in general, and to the Committee of Arrangements in particular, the thanks of the Association are most justly due for their great kindness and liberality. We are sure that no delegate can ever forget them.

---

### APPOINTMENTS.

Dr. H. J. Bigelow has been appointed Professor of Surgery in Harvard University in place of Dr. G. Hayward, resigned.

Dr. L. P. Yandell has been transferred from the chair of Chemistry, to fill the vacancy caused by the resignation of Dr. Caldwell, in the Medical department of the University of Louisville. The chair of chemistry has been filled by the election of Dr. Benjamin Silliman, Jr.

Dr. Elisha Bartlett has been elected to the chair of Pathology and Practice of Medicine, to fill the vacancy caused by the resignation of Dr. Drake in the same school.

MEDICAL CLASSES 1848–9.

| | No. of Students. | Graduates. |
|---|---|---|
| Harvard University, | 126 | |
| Albany Medical College, | 101 | |
| College of Physicians and Surgeons, New York, | 176 | 39 |
| Med. Department of the Univ. of the city of N. Y., | | 134 |
| University of Pennsylvania, | 499 | 190 |
| Jefferson Medical College, | 477 | 188 |
| Medical Department of Pennsylvania College, | | 36 |
| Philadelphia College of Medicine, | 91 | 21 |
| University of Maryland, | 190 | 64 |
| Medical College of Georgia, | 133 | 36 |
| Med. Department of Transylvania College, Lex. | 120 | 46 |
| Medical Department of University of Louisville, | 331 | 81 |
| Medical College of Ohio, Cincinnati, | 175 | 50 |
| Rush Medical College, Chicago, | 100 | 18 |
| Medical Department of University of Missouri, | 154 | 38 |
| Starling Medical College, Ohio. | 173 | 50 |

PROGRESS OF THE CHOLERA.

From our exchanges we glean the following statistics of the progress of this disease :—

At New York the official report of Saturday says that no case had occurred within 48 hours. The Board of Health have taken possession of the third story of Monroe Hall, (a very large and airy room) corner of Pearl and Centre streets, and the patients remaining at the old Ball Court, fitted up as a temporary hospital, in Anthony street, were removed on Friday evening to it.

At Maysville on the 15th the Board of Health reported no case of cholera since the 12th, yet the papers give eight or ten cases for the twenty-four hours previous, but no deaths.

At Cincinnati, same date, the official report shows 25 cases and 3 deaths—on the previous day there were 46 cases from the 12th to the 14th, and seven deaths. Reports show that from Wednesday, May 2d, up to Tuesday noon, May 15th, there occurred 314 cases of cholera, and 71 deaths by this disease in Cincinnati.

The officers of the steamer America, which arrived at St. Louis on the 9th from New Orleans, report having hired twenty or twenty-one persons who died of the cholera during the trip. Several boats have arrived at various points on the Ohio, reporting one or two deaths each.

The St. Louis Republican, of the 9th, gives a weekly summary of the deaths in St. Louis—78 out of 130 from cholera.

In considering that the cholera morbus is still increasing, the Vicar

General of the Diocese of St. Louis grants a dispensation to the Catholics of this Diocese to make use of flesh meat on Fridays, and on all days of abstinence, until the cessation of the disease.

At Louisville, on the 14th inst., four new cases of the cholera were reported and one death—a negro. The disease is still confined to certain unhealthy localities, the city generally being healthy. The whole number of deaths there since the cholera commenced does not exceed thirty.

The Norfolk Board of Health have published a report, stating that nine cases of cholera morbus had occurred in that city within the last fifteen days, five of which had terminated fatally.

A few deaths have occurred on the steamboats that have arrived from below. The Lafayette had only one death from cholera—the person came on board sick; the Magnolia had also one death; the Niagara, from St. Louis, had one death; the Diadem, from St. Louis had two deaths. The second engineer of the Paris, from St. Louis, was dying when the boat left here, and the Colorado, from Nashville, had one death.

At Chicago, the first case of cholera appeared about the 2nd of this month, and from that time to the present, there have been twenty cases and twelve deaths.—*North American and U. S. Gazette, May 22.*

